# Design and Integration of Alert Signal Detector and Separator for Hearing Aid Applications

**DOI:** 10.1109/access.2020.2999546

**Published:** 2020

**Authors:** GAUTAM SHREEDHAR BHAT, NIKHIL SHANKAR, ISSA M. S. PANAHI

**Affiliations:** Department of Electrical and Computer Engineering, The University of Texas at Dallas, Richardson, TX 75080, USA

**Keywords:** Alert signals, convolutional-recurrent neural networks (CRNN), detection, separation, speech enhancement (SE), hearing aid (HA), smartphone, real-time

## Abstract

Alert signals like sirens and home alarms are important as they warn people of precarious situations. This work presents the detection and separation of these acoustically important alert signals, not to be attenuated as noise, to assist the hearing impaired listeners. The proposed method is based on convolutional neural network (CNN) and convolutional-recurrent neural network (CRNN). The developed method consists of two blocks, the detector block, and the separator block. The entire setup is integrated with speech enhancement (SE) algorithms, and before the compression stage, used in a hearing aid device (HAD) signal processing pipeline. The detector recognizes the presence of alert signal in various noisy environments. The separator block separates the alert signal from the mixture of noisy signals before passing it through SE to ensure minimal or no attenuation of the alert signal. It is implemented on a smartphone as an application that seamlessly works with HADs in real-time. This smartphone assistive setup allows the hearing aid users to know the presence of the alert sounds even when these are out of sight. The algorithm is computationally efficient with a low processing delay. The key contribution of this paper includes the development and integration of alert signal separator block with SE and the realization of the entire setup on a smartphone in real-time. The proposed method is compared with several state-of-the-art techniques through objective measures in various noisy conditions. The experimental analysis demonstrates the effectiveness and practical usefulness of the developed setup in real-world noisy scenarios.

## I. INTRODUCTION

There are a variety of sounds produced in the environment. The range of environmental sounds includes the sounds created indoors and outdoors. Usually, such sounds convey information about surrounding environmental activities. In environmental sounds, alert signals like sirens from emergency vehicles or alarms from home security systems have high importance as they forewarn people of cautious and life-threatening situations. In adverse noisy environments, even a normal hearing individual can miss these critical warning signals leading to hazardous situations. The perception of the alert sounds becomes extremely difficult for hearing impaired listeners especially when the signals are mixed with various kinds of background noise and when they are out of sight. National Institute on Deafness and other Communication Disorders (NIDCD) reports that there are over 360 million people worldwide, including 15% of American adults i.e. about 37 million, suffering from hearing loss of some kind [1]. Personalized hearing devices like hearing aid devices (HADs) and cochlear implants (CIs) have been developed by researchers and manufacturers to improve hearing capabilities of impaired people. Developments have been made to improve the speech perception of the hearing aid (HA) users through noise suppression and speech enhancement (SE) techniques [2]. While hearing impairment is one of the most common physical disabilities in the world, little work has dealt with the role of alert sounds for people with listening impairment.

The HAD signal processing pipeline has several important modules. Acoustic feedback cancellation [3], [4], speech source localization [5], [6], SE [7]-[9], dynamic range compression (DRC) [10], [11] are some of the fundamental modules in the pipeline. SE is a vital module in the HAD signal processing pipeline as it tries to suppress the noise and enhance the performance of HADs by improving the speech quality and intelligibility perceived by people with hearing loss. Extensive studies can be found in which SE algorithms are developed to improve the efficiency of HADs in the presence of background noise. SE algorithms proposed based on statistical models [12], [13] have been effective in reducing noise at a higher signal to noise ratio (SNR) levels. There are some computationally efficient SE methods [14], [15] that work in real-time. Microphone array based SE methods [16], [17] have also worked with HADs. However, these methods achieve better performance at the cost of higher computational complexity. Recently, SE based on deep neural networks (DNN) have been proposed by researchers [18]-[21]. In the aforementioned methods, a model based on supervised learning is trained to estimate clean speech features from the noisy speech features. These DNN based approaches are known to have superior performance by achieving better noise suppression. However, the primary objective of all these methods is to suppress the background noise without causing any speech distortion. Most of the SE algorithms are application specific. The presence or the effect of the alert signals are not considered when SE algorithms are developed. Therefore, SE algorithms could mostly consider alert signals as a type of background noise and tend to attenuate these critical sounds especially when these are mixed with other environmental noise and last for rather long period of time. Although the human brain can identify specific sounds as alert sounds even if it is heard for the very first time, it becomes very difficult for HA users to identify the alert sounds when the signals are attenuated or when the source is unseen.

Research shows that hearing aid (HA) users want to be aware of different environmental sounds at all places [22]. The lowered interaction and the auditory cues from the environment can lead to a feeling of reduced safety for people with hearing impairment [23]. For example, in situations where HA user is driving a car. In such cases, the HA user may be unable to hear the emergency vehicle approaching nearby when there is high background noise or if it is attenuated. People with hearing loss will feel more safe if they are cautioned about the warning sounds and it would be even better if the warning sounds are perceived well. In literature, there are some works to detect the alert signals and thereby enhance environmental awareness. In [24], a simulated environment is generated and a set of pre-selected alarm sounds are detected through cross-correlation techniques. Artificial neural network (ANN) based pattern matching technique was used to detect police vehicle sirens in [25]. In [26]-[28] we can see works on detecting the sirens of emergency vehicles like ambulance and police cars. In [29], an alarm sound detector based on support vector machine was proposed that is tested using several audio features. A simple siren detection system that runs in real-time is described in [30]. Recently, in [31] authors proposed a warning sound detector working on a mobile platform. However, most of the methods mentioned focus only on particular type of alert signals and do not generalize. Some of the above mentioned methods do not consider frequency shifts of certain alert signals due to the Doppler effect and are tested in controlled environment like the laboratories or simulations. The majority of the methods do not have feasible solutions on how to transmit the alarm detection information to the HA user. Most importantly, these methods only consider the detection of the alert signal and do not take separation of it from the noisy speech into account. Therefore, it becomes highly improbable to incorporate many of these methods into the HAD pipeline which has noise suppression and SE modules in it. Thus, we need a better system to improve the surrounding awareness of hearing impaired people in real-world noisy environments.

**FIGURE 1.**
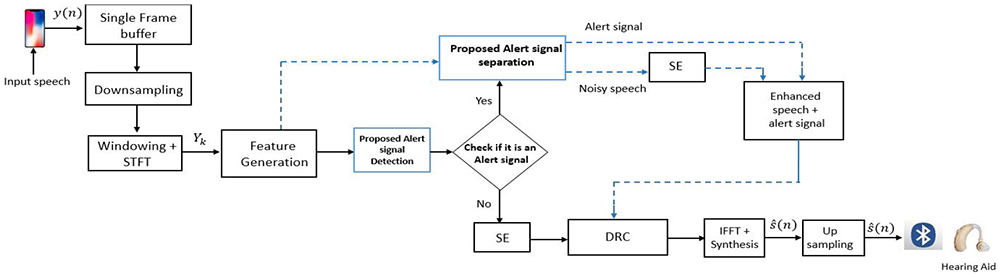
Block diagram of the proposed setup involving signal detector, separator. SE is integrated with the two blocks.

In this paper, we present a smartphone assistive setup that enhances the perception of alert signals for the HA users in noisy environments. The proposed alert signal detector and separator modules are based on convolutional neural network (CNN) and convolutional-recurrent neural networks (CRNN) respectively. We propose to use the real and the imaginary parts of the frequency domain signal as the input features for both the models. The convolutional layers extracts the information of the local patterns in input features and the recurrent layers maps the correlations between the consecutive frames. This joint optimization for the considered features improves the performance of the entire setup. The proposed method works in conjunction with SE modules used in HADs. The developed method works as an application on a smartphone in real-time that can be used as an assistive tool for hearing impaired listeners. We use a smartphone-based platform for integrating and running indispensable signal processing algorithms in real time to assist hearing impaired users. This is because it is impractical to do the same on HAD due to its limitations in size and processing capabilities. Smartphones have built-in, efficient ARM multi-core processors and sufficient resources to even run complex machine learning algorithms with low power consumption. Most importantly, smartphones are pervasive and are one of the most widely used devices everywhere. In the proposed approach, the smartphone captures the noisy speech signal comprising of alert signals, background noise and speech. The CNN based alert signal detector continuously monitors the presence of any emergency sound. If the alert signal detector detects any emergency sound, the detection is displayed on the smartphone application. The CRNN-based alert signal separator separates the alert signal from the mixture of noisy speech before passing it through SE module. The input to the SE module now contains only the speech mixed with background noise. Once the SE module is executed, the enhanced speech along with the separated alert signal goes to compression stage, and the final processed output is sent from the smartphone to the HAD through a wired connection or wirelessly via Bluetooth low energy (BLE) [32]. The proposed setup (Figure 1) ensures that there is no attenuation and/or over-amplification of the alert signal, while the alert signal detection is shown to the user on the display panel of smartphone application. The novel contribution in this work is the high performance realization and operation of the alert signal detection and separation blocks and their integration to the SE module. To the best of our knowledge, there are no published works where there is an entire setup with an alert signal detector and separator combined with the SE module of HADs. Furthermore, the whole setup is implemented on a smartphone working with low latency in real-time. The objective evaluations show the overall benefits and usability of the proposed setup for end-users.

The remainder of this paper is organized as follows. In Section II, we describe the signal model, the features used in the proposed algorithm and the developed architectures for the alert signal detector and separator. Analysis and experimental results are presented in Section III. Section IV describes the real-time implementation of the developed method on smartphone. Conclusion is in Section V.

## II. PROPOSED ALERT SIGNAL DETECTION AND SEPARATION

In this section, we discuss the signal model, the primary features, alert signal detection block, the separation block and its integration to SE module of the HAD processing pipeline. The block diagram of the proposed method is shown in Figure 1.

### A. FORMULATION AND INPUT FEATURES

Speech processing applications like speech enhancement (SE) and dynamic range compression (DRC) usually consider additive mixture model for noisy speech *y*(*n*), with clean speech *s*(*n*) and noise *v*(*n*).

(1) y(n)=s(n)+v(n)

We have to note that the noise *v*(*n*) can be mixture of background noise *d*(*n*) and alert signal *w*(*n*). The input noisy speech signal is transformed to frequency domain by taking short time Fourier transform (STFT).

(2) $$Y_{k}(\lambda )=S_{k}(\lambda )+V_{k}(\lambda )$$

*Y*_*k*_(*λ*), *S*_*k*_(*λ*), and *V*_*k*_(*λ*) represent the noisy *k*^*th*^ STFT coefficient of *y*(*n*), *s*(*n*) and *v*(*n*) respectively for frame *λ*. *k* = 0, 1, … , *N* − 1 where *N* is the STFT size.

The proposed method is based on supervised learning. It has two stages; training and testing/inferencing. Offline training is executed to generate a model and this pre-trained model is implemented on a smartphone in real-time. For both the stages, the features remain the same and the choice of the features is crucial in determining the performance of the method. A wide range of options are available to parametrically represent the speech signal. Ideal binary mask, Log power spectrum, Mel filterbank energy, Gammatone frequency power spectrum [33] are some of the widely used speech features. But, for alert signals the characteristics are different. The selection of the features for these signals plays a critical role in developing a detection model. Time domain features like pitch, Zero crossing rate (ZCR), short time energy and frequency features like spectral flux, spectral centroid, Mel frequency cepstral coefficients (MFCC) etc. have been used to recognize the warning signals [29]. However, some of the aforementioned features are not efficient in terms of computational and space complexity. Importantly, these features can only be used for signal detection and not for separation task. The alert signal separator reconstructs the signal and the above-mentioned features cannot be used for signal reconstruction (alert signal separation will be explained later in this section). In the proposed approach, we consider real and the imaginary values of the STFT of the signal as the input features. The choice of the input features is based on the fact that the trained model can learn better by using the raw STFT feature than other hand-crafted features [5]. By considering these features, we focus on both the magnitude and phase of the input which provides more information about the signal. The STFT coefficients are easy to compute and does not add much delay to input/output (i/o) latency. This is significant as it reduces complexity specifically during real-time processing. The real and the imaginary parts of the *Y*_*k*_ (*λ*) are considered as the input features for the proposed method. The following matrix shows the input feature sets.

(3) $$\left[\begin{array}{c} Real\;part\;of\;\;Y_{k=0}(\lambda )\\ \vdots \\ Real\;part\;of\;\;Y_{k=N∕2+1}(\lambda )\\ Imag.\;part\;of\;\;Y_{k=0}(\lambda )\\ \vdots \\ Imag.\;part\;of\;\;Y_{k=N∕2+1}(\lambda ) \end{array}\right]$$

Since Fourier transform of a signal is symmetric in the frequency domain, we consider only the first half of STFT of the data. Therefore, there are 2 × (*N*/2 + 1) number of real and imaginary values for every frame of STFT. The dimension of input feature set per time frame *λ* is, 1 × *F* where *F* = 2 × (*N*/2 + 1).

### B. CNN FOR ALERT SIGNAL DETECTION

A classification model that recognizes the presence of an alert signal is designed using a convolutional neural network (CNN). The proposed method is formulated as a classification problem as there are two output classes i.e. ‘alert signal-only’ and the other class is ‘no-alert signal’. A typical CNN architecture consists of convolutional, pooling and dense or fully connected layers as their hidden layers to learn complex relationships between input features and the output label. When operated for audio related works, CNNs consider a matrix as input, the hidden layers learn critical time-frequency auditory features and finally are mapped to output labels through activation functions [20].

**FIGURE 2.**
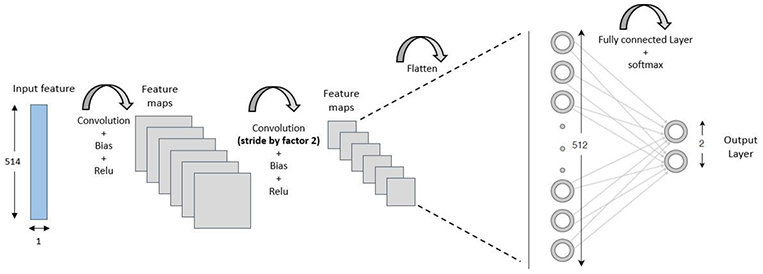
Convolutional neural network architecture for the proposed alert signal detector.

Figure 2 shows the topology of the proposed CNN-based alert signal detector. The proposed CNN architecture has 3 hidden layers, 2 convolutional and 1 fully connected (FC) layer. The input layer consists of the input feature sets explained in the previous section. We have a single dimension matrix of size 1 × *F* consisting of real and imaginary parts of STFT of the signal as input to the network. The input features are processed by the convolutional layer. In the convolutional layer, a set of learnable filters (known as kernels) are convolved with small parts of input matrix. The kernels are repeated over the entire input space. The convolutional kernels of size 5 × 1 learn the local patterns from the input features in small windows of two dimensions. In the convolutional layer, each kernel generates a 2D feature map. We apply *γ* separate filters to generate a collection of feature maps. Instead of using pooling layers, which are usually used for dimensionality reduction, the convolution operation is carried on with the stride of size 2 in the proposed approach. This makes the network computationally efficient without losing much of prediction accuracy. The feature maps are flattened before feeding to the FC layer. Rectified linear Unit (ReLU) [34] is used as activation function in convolutional layers to learn non-linear, and complex mapping between the input features and the output labels. The selection of the ReLU function is also based on its advantages of solving vanishing gradient problems.

Relu(a)=max{a,0}

The FC layer performs classification using *Softmax* activation function [35]. The softmax activation function gives the probability of each class and the one with the maximum probability is selected as the output class. The architecture of the proposed alert signal detector includes 2 convolution layers. Each convolution layer has 64 filters (*γ*) with size 5 × 1. There is one FC layer with 512 nodes. We have 2 output classes with *Softmax* activation at the output layer. The CNN model receives real and imaginary parts of STFT as inputs and generates classification results based on the presence of alert signal.

$$\beta_{i}=argmax\{p\;(\beta_{c}\;|\;\phi_{i}\},\;\;c\in (0,C-1)$$

*β*_*i*_ denotes the estimated output class i.e. the input frame is alert signal or not an alert signal, *p*(.) is the probability of *c*^*th*^ class when given the *i*^*th*^ time frame *ϕ*_*i*_. *C* is the number of output classes, which is two in the proposed case.

Since the alert signal detection block should continuously check the warning sounds in real-time, the CNN architecture is considered for detection block. CNNs are simpler than other deep learning methods. They are computationally less complex with a fewer number of parameters. This is important especially when these models have to be deployed on edge devices.

### C. ALERT SIGNAL SEPARATION USING CRNN

A regression based mapping network is developed for the proposed alert signal separation technique. The real and the imaginary values of the STFT of the siren signal is estimated by the proposed convolutional-recurrent neural network (CRNN). The alert signal separation is formulated as regression problem, as it involves reconstruction of the warning signal. A regression model is trained to estimate the features of the siren signal from the noisy input features. The input features to the proposed network are the same inputs as explained in the detector section i.e. the real and the imaginary parts of the STFT of the noisy signal, shown in Eq. (3). We consider the same features as that of the signal detector as the STFT of the raw input signal as they include spatial and temporal characteristics of a signal [36]. This also makes sure that there is no additional delay to the setup as the features will be already created in the alert signal detector block. Importantly, the real and the imaginary parts in the STFT of the signal have all the information that can be used to reconstruct the signal back to the time domain. The output labels (features) for the alert signal separator are the real and the imaginary parts of the STFT of the alert signal. CRNN acts as a mapping function between the input and the output features. Let *W*_*k*_(*λ*) be the *k*^*th*^ STFT coefficient of the alert signal *w*(*n*). *k* represents the frequency bins *k* = 0, 1, … , *N* − 1 where *N* is the size of STFT. Therefore, the output labels for the proposed architecture is given by,

(4) $$\left[\begin{array}{c} Real\;part\;of\;\;W_{k=0}(\lambda )\\ \vdots \\ Real\;part\;of\;\;W_{k=N∕2+1}(\lambda )\\ Imag.\;part\;of\;\;W_{k=0}(\lambda )\\ \vdots \\ Imag.\;part\;of\;\;W_{k=N∕2+1}(\lambda ) \end{array}\right]$$

#### Convolutional Layers:

As explained in the previous section, only the positive half of the STFT of the signal is considered due to symmetry. The dimension of the output labels is equal to input dimension i.e. (1 × *F*) where *F* = (2 × *N*/2 + 1).

**FIGURE 3.**
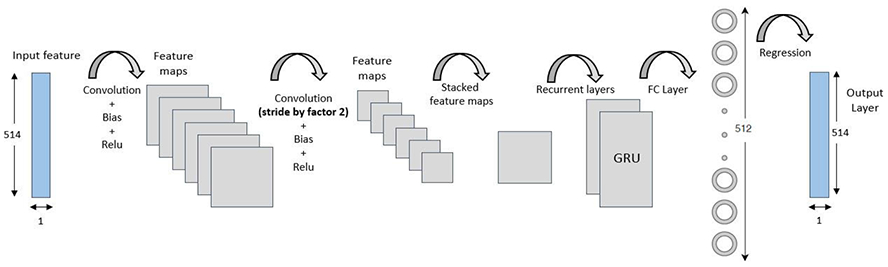
Convolutional-recurrent neural network architecture for the proposed alert signal separator.

Figure 3 shows the architecture of the CRNN based alert signal separator. There are 4 hidden layers in the topology viz. two convolutional layers, a single recurrent neural network layer (RNN) and a fully connected (FC) layer. The input layer consists of the features with size (1 × *F*). The convolutional architecture is similar to the detector block. The two convolutional layers with 64 filters are used to generate the feature maps. The kernel size is set to be (5 × 1). Due to the local similarities in the adjacent frequency bins, we propose to use stride of size 2 to perform convolution. This would considerably reduce the dimension i.e. the number of parameters and complexity in the following recurrent layer, without significant loss of accuracy. In order to reconstruct the estimated alert signal, we need to ensure the input and the predicted output to have same length in time dimension. Zero padding is applied to the input before convolution. This assures that the generated feature maps and the input are of the same dimension. The ReLU activation function is considered for the convolutional layers. We note that the advantage of using the convolutional layers in the architecture is the layers learn the specific and non-linear local patterns from the input features.

#### Temporal Learning using GRUs:

**FIGURE 4.**
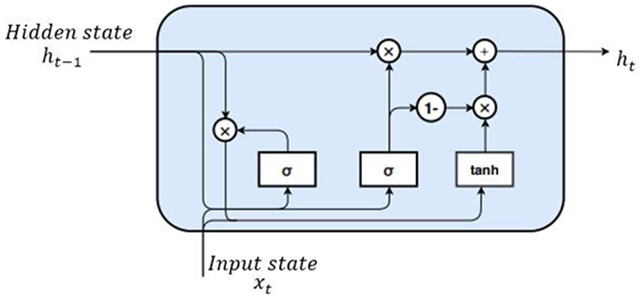
Conventional GRU cell.

Usually, the alert signals are periodic and have longer duration. Therefore, to learn the correlation between the adjacent frames, we use RNNs. The RNNs accounts for the temporal dynamics of the alert signals. In the proposed method, we stack two Gated recurrent units (GRUs) to form a recurrent layer. GRUs are a type of RNN which are capable of extracting dependencies of various time scales by recurrent units that can been applied effectively to sequential or temporal data [37]. These have been widely used in speaker recognition, language modeling etc., [38]. The GRUs have special gates to learn the relevant information in the data and increase the efficiency of learning. Figure 4 shows the GRU cell and the forward propagation of the basic GRU cell is given by,

(5) $$z_{t}=\sigma \;(G_{xz}\;x_{t}+G_{hz}\;h_{t-1}+b_{z})$$

(6) $$r_{t}=\sigma \;(G_{xr}\;x_{t}+G_{hr}\;h_{t-1}+b_{r})$$

where, *x_t_* is the input state, *z_t_* is the update gate, *r*_*t*_ is the reset gate, *h*_*t*−1_ is the hidden states at time *t* − 1 (previous state), *b*_*z*_ and *b*_*r*_ are the biases at two gates. The update gate, *z*_*t*_, aids to determine how much of the past information (from previous time steps) needs to be passed to update the hidden states. The reset gate, *r*_*t*_, is degree to forget the previous hidden state information. The gate mechanism in GRUs is used to modulate the flow of information within the unit. The *G* terms denote the weight matrices i.e *G*_*xz*_ is the weight matrix between input state and update gate, *G*_*hz*_ is the weight matrix between hidden states and the update gate. *σ* is the non-linear activation function which are be used to switch on or off the two gates.

σ(a)=1∕(1+exp(−a))

In Eq. (7), $h_{t}^{\prime }$ is known as the candidate hidden state which can be viewed as the current memory content in the GRU cell. The reset gate is used to remove the information from the previous time steps and store the relevant information from the past. ⊙ indicates an element-wise multiplication.

(7) $$h_{t}^{\prime }=tanh(G_{xz}\;x_{t}+G_{hh}\;(r_{t}\;⊙\;h_{t-1})+b_{h})$$

(8) $$h_{t}=z_{t}\;⊙\;h_{t-1}+(1-z_{t})\;⊙\;h_{t}^{\prime }$$

(9) $$y_{t}=h_{t}$$

The *tanh*(.) is an activation function, given by,

$$tanh(a)=\frac{e^{a}-e^{-a}}{e^{a}+e^{-a}}$$

**FIGURE 5.**
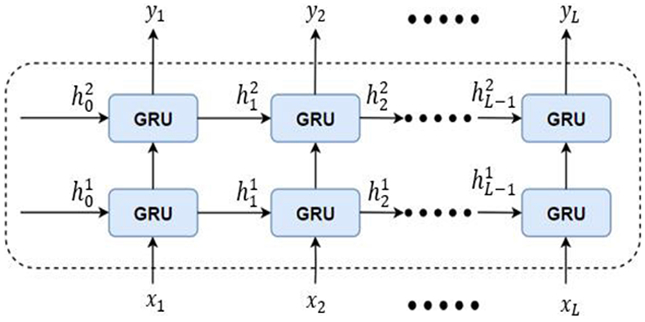
The structure of stacked GRU. The two layered stacked network is used in the proposed method.

After transforming the update gate and the reset gate, the final memory at the current time step is given by *h*_*t*_. The update gate controls the *h_t_* which holds information for the GRU cell at current time step and passes it down to the network. The model can learn to set the update gate values *z*_*t*_ close to 0 or 1. If *z*_*t*_ is close to 0, majority of the previous information is not passed to the output state. When *z*_*t*_ ≈ 0, 1 − *z*_*t*_ will be close to 1 which can be observed as, big portion of the current information is relevant to the output state at the current time step. In order to aid efficient temporal feature extraction, we use stacked GRU, which is composed of several GRU cells as shown in Figure 5.

In the proposed method, we use two stacked GRUs to form a recurrent layer. The recurrent layer is inserted between the convolutional layer and the FC layer (shown in Figure 3). We note that after the convolutional layer, the feature maps are aggregated across feature dimension to form the stacked 2D feature maps. The GRU layer has 100 cells each and the FC layer is composed of 512 nodes. The output layer has *F* nodes which is equal to the size of the input feature vector. Linear activation function is applied at the output to map the predicted output features. The CRNN uses mean squared error as the target loss function. The architecture utilizes Adadelta [39] optimization with scheduled learning for training the model.

### D. INTEGRATION TO SPEECH ENHANCEMENT

Speech enhancement (SE) is a vital component in Hearing Aid Devices (HADs). SE improves the quality and intelligibility of speech in the presence of background noise. Traditional SE algorithms are modeled considering speech to be the signal of interest and the rest of the signals in the additive mixture to be noise. Typically, in conventional SE algorithms, speech is detected by a voice activity detector (VAD) or by statistical probabilities and the noise is suppressed based on developed gain function. The warning signals which usually do not contain speech, are attenuated by SE algorithms. Recent neural network-based SE methods consider clean speech features as their output label to develop a neural network model. These SE methods tend to distort the signals when warning signals are present in an unseen environment. This performance is expected as the researchers do not consider the presence of these critical signals while training the SE model. Therefore, through our experiments, we observed that most of the SE algorithms either attenuate the alert signal or the processed signal is distorted when there is an alert signal mixed in the background. In the proposed setup, the real and the imaginary values i.e. the input features are extracted from the input noisy signal. The developed CNN-based alert signal detection block is used before the SE module to continuously check for the presence of any alert sounds in the input signal. If there is no presence of alert signal, the signal is passed to the SE module for background noise suppression. If the alert signal is detected by the alert signal detector, the user is notified, and the same noisy input features are used as the input to the alert signal separator. The alert signal is separated from the mixture of the signals. The input noisy speech free from the alert signal is processed by the SE module. The alert signal is then be added back to the enhanced speech. This signal can be passed to other signal processing modules in the HADs or can be converted to the time domain by taking inverse Fast Fourier Transform (IFFT) of the signal. The proposed setup ensures that there is no attenuation of the alert signal and no distortion in the processed speech. The setup overcomes the constraints of losing the information in emergency conditions for hearing impaired and even for normal hearing people. The overall pipeline of the method is shown in Figure 1.

## III. EXPERIMENTAL ANALYSIS AND RESULTS

In this section, we discuss the experimental evaluations carried out on the alert signal detector and the alert signal separator.

### A. DATASET

To train and evaluate the developed CRNN-based alert signal detector and separator, the alert signals are mixed with noisy speech files at different SNR levels. Different types of alert signals have varying characteristics. A standard for “auditory danger signals” (ISO 7731) [40] has been established by the International Organization for Standardisation. However, this provides basic instructions for warning sounds and is not commonly used around the world. In order to achieve generalization and to generate robust models, it is important to include the variety of alert signals with all the unique characteristics. In [41], the common characteristics of the alert signal are mentioned. Some of the types of alert signals are,

- Pulsed alarms - Consists of a repeat of the same sound with silence between the instances.
- Sirens - Sounds, in which the frequency varies constantly. ‘Wail’, ‘Yelp’ and ‘Hi-Lo’ are the major patterns found in sirens. Wail and Yelp are the signals in which the pitch of the signal rises and falls over time. Wail and yelp have the same basic composition. However, in Yelp, the pitch alternates rapidly. Hi-Lo is the two-tone sirens that have two signals with different frequencies.
- Alternating alarms - Consists of two distinct alternating tones with no silence between them. These can also be viewed as a type of Hi-Lo sirens.

**FIGURE 6.**
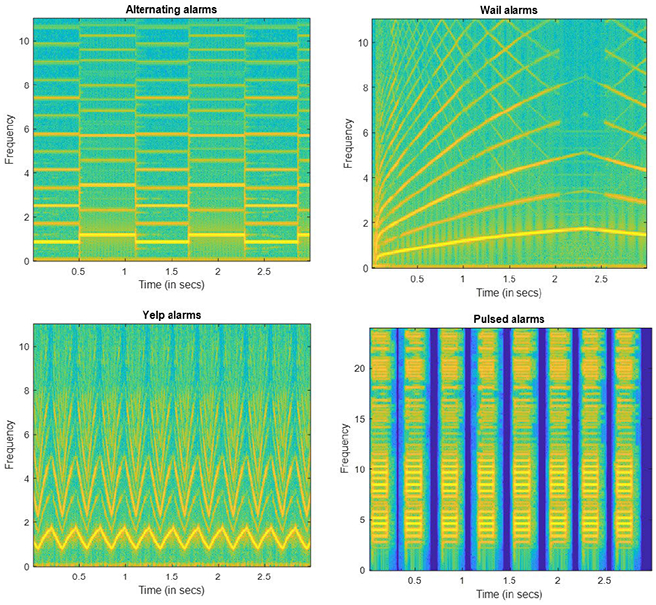
Spectrograms of different types of alarm sounds. a) Alternating alarm usually used in Fire alarms. b) An emergency vehicle driving away (Sirens). c) Yelp alarms (frequency continuously changes). d) Pulsed alarm signals.

Figure 6 shows the spectrogram of the types of alert signals considered. A large database was designed using different web sources. All the above mentioned types of alert signals were included in the dataset. We note that, signals with frequency shifts due to the doppler effect were also considered, especially for Wail and Yelp type of outdoor siren signals.

The clean speech sentences were selected from HINT, TIMIT and LibriSpeech corpus [42]. The noise files are selected from the DCASE 2017 challenge database [43]. Three major outdoor noise types machinery, traffic and multi-talker babble are considered as they are commonly seen in real-life environment. Along with this, more than 50 smartphone collected realistic noise is included in the noise database. The alert signals, speech sentences, and noise files were selected from various sources as it improves generalization. It is also important as it helps to work in real-world noisy conditions. We note that the noisy speech files were created by adding speech and noise at 0 dB SNR. The noisy speech was mixed with alert signals at SNR levels from −5 dB to +10 dB with an increment of 5 dB. All the signals were sampled at 16 kHz. An overall of 60 hours of data was used for training. Only 30% of the database had alert signals mixed with noisy speech. This is because the amount of alert signals is extremely low when compared to the no-alert signals in real-life scenarios.

### B. OFFLINE OBJECTIVE EVALUATION FOR ALERT SIGNAL DETECTOR

**FIGURE 7.**
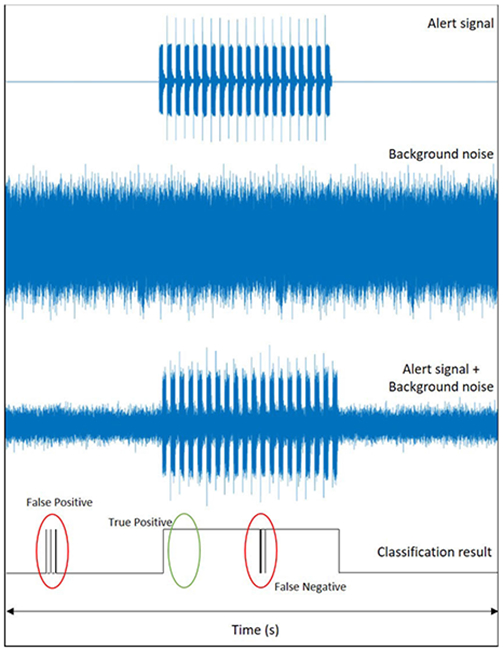
Representation of True Positives, False Positives and False Negatives considered as the objective measures for siren detection. An example of classification results for alert signal mixed with background noise is shown.

The performance of the proposed detection method is evaluated in this section. The proposed detection method is compared with two other methods. A conventional method based on autocorrelation [41] and a feed forward neural network based siren detection [31] algorithms are compared with the proposed detection technique. As performance metrics, we use true positives (TP), False Positives (FP) and False negatives (FN). TP can be viewed as the percentage of alert signal frames correctly classified. FP is the percentage of non-alert signal frames classified as alert signal frames. FN is the percentage of alert signal frames classified as non-alert signal frames. Higher TP means, higher is the accuracy of detection. It is ideal to have lower FP and FN as they indicate smaller chances of error. True Negatives (TN) are not considered in this experiment as they are considerably less significant in the proposed application. Figure 7 shows definition used for TP, FP and FN. Experimental evaluations are performed for 3 different noise types; machinery, multi-talker babble, and traffic noise. Table 1 shows the comparison of TP, FP and FN results averaged over 20 sentences. We note that, the speech signals, the noise files, and the alert signals used for objective measures are validation data i.e. the dataset was unseen by the model and were not included for training and testing. On an average, the proposed method is ≈30% and ≈13% better in true positive rate when compared to conventional and the DNN method respectively. From Table 1 we can observe that higher the SNR, lower the TP and higher the FP/ FN. This performance is expected because, as the SNR increases, the power of the noisy speech increases. The Objective measures show significant improvements over conventional and deep learning methods for all the three noise types considered.

### C. OFFLINE OBJECTIVE EVALUATION FOR ALERT SIGNAL SEPARATION

This section describes the performance evaluation of the proposed alert signal separation method when integrated with the speech enhancement (SE) techniques. The alert signal separator ensures that there is no attenuation of the warning signals. However, it is essential to guarantee that there are no distortions, and processing artifacts. It is also important to note that the entire setup does not affect the speech intelligibility. The proposed method is evaluated using a performance measures, Signal to Distortion Ratio (SDR), Signal to Interference Ratio (SIR), Signal to Artifact Ratio (SAR) [44], and Coherence Speech Intelligibility Index (CSII) [45]. The alert signal separation method can be considered as a type of single channel source separation, therefore we use the above mentioned objective measures. SIR measures the effect of other sources on the separated source and shows how much interference the other signals have on the signal of interest. SAR measures if there are any residual noise or other artifacts introduced by the proposed method. SDR measures the overall separation quality. Higher SIR, SAR and SDR measures mean the separated signal has minimal artifacts and distortion. CSII is the speech intelligibility measure which varies from 0 to 1, with 1 being high intelligibility.

To the best of our knowledge, there are no published works on alert signal separation and its integration with speech enhancement. Therefore, we compare the proposed alert signal separation method integrated with several SE methods. The conventional SE method based on Log-MMSE [13], and a convolutional neural network (CNN) based SE [20] methods are integrated with the proposed alert signal separation block to evaluate the performance. We test the results of the integrated setup because the aim of the proposed method is to ensure there is no attenuation of the alert signals and no distortion in the processed speech after SE. Machinery, Multi-talker babble and Traffic noise types are considered. The noisy speech files were created by adding speech and noise at 0 dB SNR. The noisy speech was mixed with alert signals at different SNR levels. We note that if the SNR mentioned is +10 dB, the power of the noisy speech is 10 dB higher than the power of the alert signal. As considered in the previous section for detection method comparison, the validation sentences are unseen by the model. Tables 2 and 3 show the objective results for the proposed separation method integrated with SE techniques. The results shown are the average of over 15 sentences. In the tables, the unprocessed signal is the mixture of noisy speech and alert signal. The Conv. and the CNN represent the signals processed using SE methods [13] and [20] respectively without any separation. i.e. the mixture of noisy speech and alert signal does not pass through proposed separation block and are processed using SE methods alone. Conv. + separation and CNN + separation represent the signals processed using SE methods [13] and [20] respectively with separation. i.e. the mixture of noisy speech and alert signal is processed using the proposed alert signal separation method to separate the alert signal, the estimated noisy speech free from the alert signal is processed using SE methods to generate enhanced speech. The separated alert signal is added back to the enhanced speech.

Objective measures show significant improvements over conventional and deep learning method for all three noise types considered. From the Tables 2 and 3 we observe that on an average, the inclusion of the proposed separator block increases the SAR and SDR ≈5.05 dB. The SIR also increases by ≈6.18 dB. This shows that the overall quality of the output signal improves significantly while preserving the alert signals. The proposed setup also improves the speech intelligibility. Table 3 shows the CSII results at different SNRs for three different noise types. This shows that the addition of the alert signal separator block does not degrade the intelligibility of speech. Objective measures shown in Tables 2 and 3 reemphasize the fact that the proposed method achieves comparatively more noise suppression without attenuating the warning signals and without distorting speech.

### D. SCALING NETWORK FOR SEPARATION BLOCK

The proposed CRNN architecture is scaled by controlling the number of trainable parameters. The proposed architecture is scaled to have total of a 3, 9, 15, and 27 million parameters with a tolerance of 5%. Considering the limitations like latency, accuracy, training time and the hardware capabilities, through our experiments we consider the upper bound to be 27M parameters. The size of the model becomes significant when it is used to deploy on edge devices (example: smartphones, laptops, raspberry pi, etc.). This experiment gives an overview of how the performance of the model varies for the architecture with the same depth but the different number of hidden units. Table 4 summarises the details of different structures including the layer width (the number of feature maps). The width of each layer is changed to control the number of parameters. We note that the depth of the architecture is the same. This ensures that the hierarchy of the learned features remains the same and only the number of features in each layer changes. The size of the convolutional kernel, the stride size, the training batch size are set to be the same. Table 5 shows the comparison of the objective measures used for the scaled CRNN network. The results are shown for alert signals mixed with noisy speech (traffic noise) at 0 dB. We consider traffic noise type in this experiment as it was considerably more challenging than the others. From the table, we can observe that as expected, the objective results were comparatively better as the number of learnable parameters increased. However, the proposed architecture with ≈9M parameters had a better trade-off with the performance of alert signal separation and computational complexity. Neural network models under 10M parameters have been implemented on edge devices like smartphones [20]. Considering these parameters, the model with 9M parameters is used for smartphone implementation. The same model is used for the objective results shown in Tables 2 and 3. Models with a higher number of parameters can also be implemented on edge devices that have high computational capabilities.

### E. COMPARISON OF GRU WITH LSTM

The efficiency of GRUs is compared with LSTMs (Long Short Term Memory units) in this experiment. LSTMs are a type of recurrent neural network which also use gating mechanism to control the flow of information to the current hidden units. LSTM cells have four gates to transfer the information which is two more than GRUs. Two different models are trained and evaluated. The proposed CRNN model with stacked GRU cells as the RNN layer. The GRUs are replaced with the stacked LSTM cells to compare the performance. The rest of the network architecture remains the same. The input and the output features are the same i.e. the real the imaginary parts of the FFT of the input signal. Table 6 shows the performance of the proposed setup with GRU and LSTM networks. The clean speech is mixed with different noise types at 0 dB SNR and the noisy speech is at 0 dB SNR with respect to the alert signal. The performance of the two networks integrated with conventional SE technique is shown in Table 6. The results suggest that the proposed GRU model performs slightly better than that of the LSTM model. The additional gates in the LSTM network increase the number of learnable parameters by ≈10%. Thus, the cost of computations and complexity increases. Because of the following limitations and degradation, in the proposed method we considered GRUs over LSTMs.

### F. UNSEEN SNR EFFECT

In this experiment, we assess the influence of the unknown SNR on the proposed model. The changes in the SNR is common and often rapid in real-world noisy environments. So, we need a robust model that will be able to overcome these rapid SNR shifts in real time. To examine the effect of unseen SNR, the proposed CRNN models trained at −5 dB and +10 dB SNRs are tested with signals at different SNR. We consider the two conditions as they are extreme cases where the power of the alert signal is 5 dB higher than the noisy speech and +10 dB lower than the noisy speech. The model trained at −5 dB SNR is tested with the unseen signals at an unseen SNR of −10 dB (the power of the alert signal is 10 dB higher than the power of the noisy speech). Similarly, the model trained at +10 dB SNR is tested with the unseen signals at an unseen SNR of +15 dB (the power of the noisy speech is 15 dB higher than the power of the alert signal). Table 7 shows the performance evaluation of the proposed integrated setup tested at unseen SNR conditions. Clean speech degraded by traffic noise at 0 dB SNR is used as noisy speech to evaluate the performance of the proposed method in unseen SNR. For comparison, we use unprocessed noisy speech mixed with the alert signal, the signals processed using only conventional SE method and the signals processed using integrated setup of CRNN separation and conventional SE method. From Table 7 we can see that, even under unknown SNR conditions the proposed setup out-performs other methods. The trends were similar with other noise types like multi-talker babble and machinery noise. The results shown in Table 7 indicate that the model can be used in realistic environments with unknown and changing SNR conditions.

## IV. REAL-TIME IMPLEMENTATION ON SMARTPHONE

In this section, we discuss the steps and tools involved in the real-time implementation on smartphone. As an example, we choose iOS-based smartphones (iPhones) as our implementation platform. However, the proposed method can work seamlessly on android devices or other edge devices like laptops. The video demonstration of the proposed method running on a smartphone can be seen in [46].

### A. OFFLINE TRAINING AND TOOLS

The models used for smartphone implementation are trained offline. For training the detection and the separation model, the input features i.e. the real and the imaginary parts of the STFT are generated using MatLab. For input data generation, each input data frame of the noisy speech signal mixed with the alert signal is sampled at 16kHz. Each frame input data of size 32ms with a 50% overlap is windowed using the Hamming window. An STFT size of 512 (*N*) is considered to generate the real and the imaginary parts of the STFT. Therefore, 257 (*N*/2 + 1) real and imaginary parts of the STFT form the input features. Therefore, the dimension of the input data will be 514 for each frame. The output labels for the detection and the separation models are generated in MatLab. After data generation, GPU and cloud-based training are employed for generating the detection and separation models. Tensorflow software [47] is used for model design and offline training. Tensorflow is considered for training as it provides framework called Tensorflow-Lite (tflite) [48] for implementing deep learning models on edge devices. Tensorflow-Lite provides a library called tflite Converter to convert trained models to (.tflite) version. These models in (.tflite) versions are optimized to be used as inference-only models on mobile and embedded devices that have limited resources. Firebase software development kit (SDK) [49] is used to provide custom APIs which are added to the iOS application. These APIs help to provide on-device model inference. The feature extraction and SE on smartphone application were coded in C++. Xcode [50]was used for coding and debugging. Objective C was used for on-device inference and GUI deployment. Core Audio framework [51], is used to carry out input/ output (i/o) handling for audio processing. We note that all software tools and frameworks used are open source.

### B. REAL-TIME PROCESSING

**FIGURE 8.**
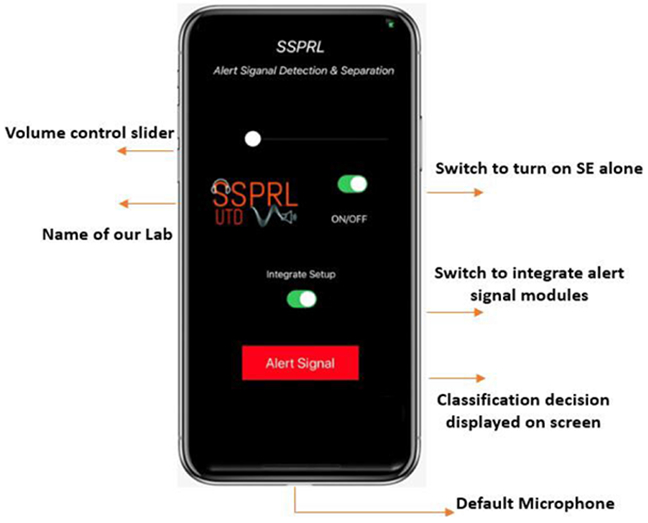
GUI of the developed smartphone application running on an iPhone.

The proposed set-up can work as a real-time application on any ARM processing platform. In the proposed method, we consider iPhone 11 smartphone running on iOS 13.1.1 for real-time implementation. For real-time processing, the entire setup that includes alert signal detection and separation integrated to the SE module is implemented on a smartphone. Input data is captured on the smartphone with a frame size of 32ms with an overlap of 50% at a 48 kHz sampling rate. The captured data is downsampled to 16 kHz by low-pass filtering and a decimation factor of 3. Therefore, there are 512 samples (32ms in time) for every processing frame frame. A 512 point STFT is computed and only the first 257 (*N*/2 + 1) real and imaginary values are considered. The input feature vector of size (514 × 1) is computed. This feature vector is continuously fed to the pre-trained CNN-based detection model. The output of the detection model is the classification output which detects the presence of the alert signal. The detection model works for every frame to monitor the presence of any warning sounds. The Graphical User Interface (GUI) is updated to display the classification result on the smartphone screen for the user as shown in Figure 8. If the 5 consecutive previous frames are classified as alert signal, the input features, are fed to the CRNN-based alert signal separation model. The output of the separation model is the estimate of the real and the imaginary parts of the STFT of the alert signal. The estimated alert signal is separated from the mixture of noisy speech and the alert signal. The estimate of the noisy speech which does not have the alert signal is then passed to the SE module for noise suppression. After applying IFFT and reconstruction, the enhanced speech and the alert signal are then transmitted to the HAD via Bluetooth low energy. When the 20 consecutive previous frames are classified as non-alert signal, the CRNN separation block is deactivated. This deactivation time is set to be large because some pulsed alert signals have large silence between the sounds. However, the detection block and the SE work continuously. In the Figure 8, the button shown on the upper part of the screen controls the application. When the button is ‘OFF’ the application acts like usual audio play-back without any processing. When the button is ‘ON’, the application works as SE alone. The conventional SE based on LogMMSE [13] is implemented as the SE module. The button on the lower part of the screen controls the alert signal detection and separation. When this button is ‘ON’ the alert signal detection and the separation block is integrated with the SE module. Once the module is integrated, the application takes approximately 1.8 seconds for Firebase to initialize the tflite inference only models. After the initialization, the application runs seamlessly in real-time. The proposed CRNN model with 9M parameters is considered for the smartphone implementation. The overall i/o audio latency of the application ≈14 ms. The i/o latency on iPhone ≈9 ms [52]. The processing latency of the entire setup is, 5.21ms. The SE alone has a processing delay of 4.2ms, the alert signal detection and the separation inference time for each frame is approximately 0.36ms and 0.65ms respectively. All these measures were calculated on the smartphone for an input frame of size 32ms. Reference [46] shows the iOS app running on iPhone 11.

### C. SMARTPHONE TESTING

In offline conditions with a controlled environment, most of the methods work extremely well. However, their performance degrades significantly when tested in real-time and under varying acoustic conditions. Smartphones are portable and can be used in challenging conditions, such as constant motion, varying SNRs or varying noise. Therefore, it is important to evaluate the real-time performance of the proposed method on a smartphone platform. In order to test the real-time operation of the proposed setup on the smartphone platform, a mixture of alert signals and known noisy speech sentences were played approximately at 0 dB SNR. These signals were processed by smartphone (in real-time) and on a PC (in offline mode). The classification and the separation outcomes of the smartphone are stored to compare with the offline method. The performance assessment in offline (PC) and in real-time (smartphone) conditions of the proposed method are shown in Tables 8 and 9. The true positives, false positives and false negative results for the alert signal classification performed in real-time and offline conditions are shown in Table 8. Table 9 shows the performance evaluation of the proposed integrated setup in real-time and offline conditions. Tables 8 and 9 show that the results measured in a real-time condition on smartphone are similar to the offline process. This experiment shows that the model performs well when tested on the smartphone platform. The sample audio files enhanced using the integrated setup can be found in [53].

### D. SMARTPHONE APPLICATION CHARACTERISTICS

**FIGURE 9.**
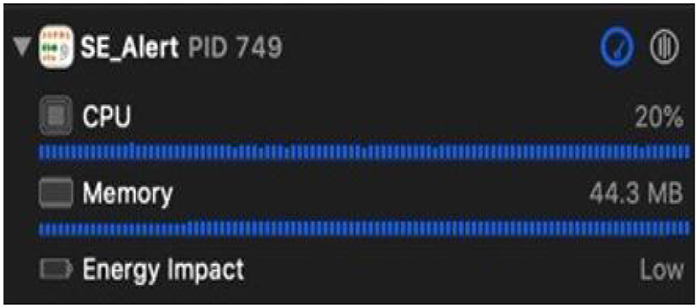
Battery, RAM and the CPU consumption of the proposed integrated setup running on a smartphone as real-time application.

In this section, we discuss the computational burden on the smartphone when the entire setup is running as a real-time app. The application’s CPU consumption is low. Even though the app makes use of the audio frame work, Firebase APIs, and inferences two neural network models, the overall CPU usage of the setup is around 19-20%. The memory used by the application is around 44.3 MB. The iPhone 11 smartphone has a RAM of size 4GB. Therefore, the app uses ≈1.1% of the memory. The memory consumption is quite low considering the tools and the computations in the app. This shows that the app will not overload the smartphone’s CPU and memory space. Since the developed application uses minimal smartphone resource, it can be used when the smartphone is running other apps in the background. The energy impact of the app is also low. The application runs about 8 hours on a fully charged iPhone 11 which has a battery capacity of 3046mAH. Figure 9 shows the CPU, memory and battery usage of the proposed application when it is running on the smartphone. While energy consumption and memory usage are both low, it is worth noting that it is better to use simple networks instead of larger networks with a higher number of parameters. That is because deeper networks typically have long inference time that can increase the overall latency in real-time.

## V. CONCLUSION

In this paper, we presented a neural network-based alert signal detector and separator. The alert signal detector is based on convolutional neural network (CNN). The separator is based on convolutional-recurrent neural network (CRNN) with stacked GRUs as the recurrent layer. The developed methods were integrated with speech enhancement techniques used in hearing aid devices. The alert signal detector and separator blocks ensure that there is no attenuation of critical warning sounds. The entire setup is implemented on a smartphone that works in real-time to improve the environmental awareness for people with hearing loss. The proposed method is computationally efficient and optimized to have minimal audio latency. The objective measures for each block of the setup affirm the usefulness and applicability of the proposed approach in various noisy conditions in the real world. The proposed setup on the smartphone provides a cost-effective and portable system that can be used by people with listening impairment, audiologists and researchers for improving the hearing study.

## Figures and Tables

**TABLE 1.** Comparison of the classification results of the proposed method with conventional and DNN based method at various SNRs and noise types. The TP, FN, FP results are in %.

Noise Type	SNR(dB)	Conventional			DNN			Proposed		
		TP	FN	FP	TP	FN	FP	TP	FN	FP
	−5	83.33	16.67	1.20	91.21	8.76	4.71	**97.60**	**2.40**	**1.02**
Machinery	0	77.00	23.00	2.14	87.80	12.20	7.10	**95.41**	**4.59**	**1.45**
	+5	70.36	30.64	3.95	79.79	20.21	8.49	**93.47**	**6.53**	**2.92**
	+10	67.23	33.77	7.46	67.25	32.75	13.23	**90.29**	**9.71**	**3.65**
	−5	76.00	24.00	3.16	91.32	8.68	5.65	**97.96**	**2.04**	**0.85**
Babble	0	72.32	27.68	6.20	87.11	12.89	8.20	**94.00**	**6.00**	**1.18**
	+5	66.79	33.21	8.69	85.64	14.36	9.44	**92.37**	**7.63**	**2.11**
	+10	55.08	44.92	10.90	83.80	16.20	14.63	**90.42**	**9.58**	**3.04**
	−5	77.22	22.78	3.21	90.02	9.98	2.16	**96.94**	**3.06**	**0.60**
Traffic	0	71.66	28.34	4.12	85.45	14.55	5.40	**94.39**	**5.61**	**1.39**
	+5	69.70	30.30	8.30	76.99	23.01	9.00	**90.66**	**9.34**	**2.08**
	+10	68.35	31.65	12.66	74.84	25.16	12.41	**89.72**	**10.28**	**2.96**

**TABLE 2.** Comparison of SAR and SDR objective measures for 3 different noise types at various SNRs. The comparison Is for unprocessed signal and for the SE methods with and without the proposed separation module.

Noise Type	SNR(dB)	Unprocessed	Conv.	SAR Conv. + separation	CNN	CNN + separation	Unprocessed	Conv.	SDR Conv. + separation	CNN	CNN + separation
	−5	−17.46	−19.52	**−14.3**	−19.63	**−14.94**	−16.71	−19.16	**−14.59**	−19.8	**−14.52**
Traffic	0	−7.54	−8.77	**−2.32**	−9.55	**−2.95**	−7.5	−8.84	**−3.18**	−9.62	**−3.61**
	+5	−5.46	−5.68	**−2.01**	−6.11	**−2.35**	−5.21	−5.48	**−1.86**	−6.16	**−2.21**
	+10	−3.9	−3.13	**−1.9**	−3.26	**−1.98**	−3.92	−3.17	**−0.90**	−3.87	**−1.23**
	−5	−13.13	−13.02	**−9.95**	−14.65	**−10.09**	−13.98	−13.1	**−9.9**	−13.07	**−9.18**
Machinery	0	−3.26	−2.96	**1.37**	−3.02	**0.95**	−4.35	−3.04	**1.32**	−3.96	**1.26**
	+5	−0.96	−0.75	**2.08**	−0.89	**1.41**	−1.55	−0.85	**2.05**	−1.39	**1.85**
	+10	1.79	2.62	**4.17**	2.38	**4.02**	1.78	2.8	**4.15**	2.09	**3.57**
	−5	−19.67	−20.3	**−14.74**	−20.82	**−15.31**	−21.21	−20.03	**−14.90**	−21.09	**−15.86**
Babble	0	−17.32	−16.57	**−4.02**	−16.24	**−4.54**	−18.13	−18.04	**−4.36**	−18.85	**−4.39**
	+5	−11.04	−10.03	**−2.83**	−10.21	**−3.43**	−11.38	−10.69	**−2.99**	−11.50	**−3.22**
	+10	−5.36	−4.05	**−1.8**	−4.74	**−2.52**	−5.42	−4.09	**−1.83**	−4.95	**−2.28**

**TABLE 3.** Comparison of SIR and CSII objective measures for 3 different noise types at various SNRs. The comparison is for unprocessed signal and for the SE methods with and without the proposed separation module.

Noise Type	SNR(dB)	Unprocessed	Conv.	SAR Conv. + separation	CNN	CNN + separation	Unprocessed	Conv.	SDR Conv. + separation	CNN	CNN + separation
	−5	14.59	9.80	**18.55**	9.44	**16.89**	0.2954	0.2905	**0.4323**	0.2860	**0.4042**
Traffic	0	21.97	19.92	**24.64**	18.49	**24.02**	0.3885	0.4045	**0.5243**	0.3890	**0.4759**
	+5	22.12	21.23	**25.15**	19.69	**24.70**	0.4030	0.4087	**0.5308**	0.3899	**0.4882**
	+10	22.72	22.96	**26.17**	21.28	**25.88**	0.4279	0.4186	**0.5430**	0.4010	**0.5078**
	−5	17.09	17.7	**20.13**	17.13	**20.65**	0.5067	0.5042	**0.5731**	0.4819	**0.5646**
Machinery	0	18.65	19.07	**23.02**	18.84	**22.43**	0.5100	0.5002	**0.6222**	0.4963	**0.5181**
	+5	22.47	23.00	**26.63**	22.52	**25.83**	0.5522	0.5764	**0.6472**	0.5055	**0.5845**
	+10	27.88	28.54	**32.12**	27.79	**31.05**	0.6331	0.6931	**0.7177**	0.6609	**0.7009**
	−5	3.67	4.05	**14.3**	3.89	**14.06**	0.2006	0.2467	**0.2793**	0.2324	**0.2758**
Babble	0	9.07	9.13	**21.86**	8.99	**20.01**	0.2746	0.3664	**0.4178**	0.3537	**0.3944**
	+5	14.20	15.31	**22.98**	14.78	**21.59**	0.3205	0.3827	**0.4354**	0.3684	**0.4162**
	+10	19.69	21.87	**24.67**	20.93	**23.70**	0.4089	0.4452	**0.5015**	0.4227	**0.4853**

**TABLE 4.** Comparison of the architecture of scaling networks. The layers considered, the width of the each layer and the total number of parameters is shown.

CRNN architecture	No. of parameters			
	3M	9M	15M	27M
Layer Type	Layer Width			
2D convolution	64	64	128	256
2D convolution	64	64	128	256
stacked GRU	70	100	150	150
Fully connected	256	512	512	1024

**TABLE 5.** Comparison of the objective measures for scaling networks. The clean speech is mixed with traffic noise at 0 dB SNR. The noisy speech is at 0 dB to the alert signals.

Scaling Network	SE Method + Separation	Objective measures			
		SAR	SDR	SIR	CSII
	Conv.	−4.50	−4.54	21.05	0.47
3M	CNN	−5.71	−5.77	20.52	0.44
	Conv.	−2.32	−3.18	24.64	0.52
9M	CNN	−2.95	−3.61	24.02	0.47
	Conv.	−2.07	−3.08	25.17	0.54
15M	CNN	−2.66	−3.48	24.9	0.50
	Conv.	−1.90	−2.91	25.72	0.54
27M	CNN	−2.36	−3.12	25.04	0.51

**TABLE 6.** Comparison of the proposed GRU model with LSTM model for different noise types. Noisy speech mixed with alert signal at 0 dB SNR.

Noise Type	SE Method + Separation	Objective measures			
		SAR	SDR	SIR	CSII
	GRU	**−2.32**	**−3.18**	**24.64**	**0.5243**
Traffic	LSTM	−2.56	−3.90	24.17	0.5135
	GRU	**1.37**	**1.32**	**24.67**	**0.6222**
Machinery	LSTM	0.94	0.90	24.09	0.6142
	GRU	**−4.02**	**−4.36**	**21.86**	**0.4178**
Babble	LSTM	−4.33	−4.72	21.08	0.4096

**TABLE 7.** Performance evaluation in unseen SNR condition for noisy speech (traffic noise at 0 dB) mixed with alert signal. The neural network models were trained at −5 dB and +10 dB SNR, tested at −10 dB and +15 dB respectively.

SNR dB	Method	Objective measures			
		SAR	SDR	SIR	CSII
	Unprocessed	−19.82	−19.32	7.21	0.2303
−10	Conv.	−19.88	−20.27	5.21	0.2309
	**Conv + Separation**	**−15.77**	**−15.93**	**9.92**	**0.3981**
	Unprocessed	−2.32	−2.36	22.27	0.439
+15	Conv.	−2.64	−2.66	22.05	0.4511
	**Conv + Separation**	**−1.71**	**−1.77**	**23.59**	**0.5334**

**TABLE 8.** Alert signal detection method tested on PC and smartphone platform. The real-time smartphone tested results are on par with the results tested offline.

Platform	Objective measures		
	TP	FP	FN
PC (Offline)	94.61	2.07	5.39
Smartphone	92.80	3.85	7.20

**TABLE 9.** Alert signal separation method tested on PC and smartphone platform. The real-time smartphone tested results are on par with the results tested offline.

Platform	Objective measures			
	SAR	SDR	SIR	CSII
PC (Offline)	−1.53	−1.94	23.29	0.6430
Smartphone	−2.65	−3.07	22.17	0.6007
